# Quantification of Tortuosity and Fractal Dimension of the Lung Vessels in Pulmonary Hypertension Patients

**DOI:** 10.1371/journal.pone.0087515

**Published:** 2014-01-31

**Authors:** Michael Helmberger, Michael Pienn, Martin Urschler, Peter Kullnig, Rudolf Stollberger, Gabor Kovacs, Andrea Olschewski, Horst Olschewski, Zoltán Bálint

**Affiliations:** 1 Ludwig Boltzmann Institute for Lung Vascular Research, Graz, Austria; 2 Institute for Computer Graphics and Vision, Graz University of Technology, Graz, Austria; 3 Ludwig Boltzmann Institute for Clinical Forensic Imaging, Graz, Austria; 4 DiagnostikZentrum Graz, Graz, Austria; 5 Institute for Medical Engineering, Graz University of Technology, Graz, Austria; 6 Division of Pulmonology, Department of Internal Medicine, Medical University of Graz, Graz, Austria; 7 Experimental Anesthesiology, Department of Anesthesia and Intensive Care Medicine, Medical University of Graz, Graz, Austria; Sapienza University of Rome, Italy

## Abstract

Pulmonary hypertension (PH) can result in vascular pruning and increased tortuosity of the blood vessels. In this study we examined whether automatic extraction of lung vessels from contrast-enhanced thoracic computed tomography (CT) scans and calculation of tortuosity as well as 3D fractal dimension of the segmented lung vessels results in measures associated with PH.

In this pilot study, 24 patients (18 with and 6 without PH) were examined with thorax CT following their diagnostic or follow-up right-sided heart catheterisation (RHC). Images of the whole thorax were acquired with a 128-slice dual-energy CT scanner. After lung identification, a vessel enhancement filter was used to estimate the lung vessel centerlines. From these, the vascular trees were generated. For each vessel segment the tortuosity was calculated using distance metric. Fractal dimension was computed using 3D box counting. Hemodynamic data from RHC was used for correlation analysis.

Distance metric, the readout of vessel tortuosity, correlated with mean pulmonary arterial pressure (Spearman correlation coefficient: ρ = 0.60) and other relevant parameters, like pulmonary vascular resistance (ρ = 0.59), arterio-venous difference in oxygen (ρ = 0.54), arterial (ρ = −0.54) and venous oxygen saturation (ρ = −0.68). Moreover, distance metric increased with increase of WHO functional class. In contrast, 3D fractal dimension was only significantly correlated with arterial oxygen saturation (ρ = 0.47).

Automatic detection of the lung vascular tree can provide clinically relevant measures of blood vessel morphology. Non-invasive quantification of pulmonary vessel tortuosity may provide a tool to evaluate the severity of pulmonary hypertension.

**Trial Registration:**

ClinicalTrials.gov NCT01607489

## Introduction

Pulmonary hypertension (PH) is a chronic disorder of the pulmonary circulation, marked by an elevated vascular resistance and pressure. This results in functional limitations, increased load on the right heart and may subsequently lead to right-heart failure [1]. PH is defined as a mean pulmonary arterial pressure (mPAP) ≥25 mmHg, which is determined during invasive right heart catheterisation (RHC) [1], [2]. Despite the low risk of adverse events of this invasive investigation, there is a need for non-invasive procedures to support the indication for an RHC investigation or to replace an invasive procedure in the follow-up of patients [3], [4].

Radiological features of PH are vascular pruning due to vascular remodelling and loss of arterial branching [5]. Recently, a non-invasive, thoracic computed tomography derived, lung vessel based diagnostic method for chronic obstructive lung diseases (COPD) was presented [6]. The authors characterize smoking-related COPD by the magnitude of distal pruning measured from automatically identified and segmented lung vessels. Application of parallel computing algorithms on general purpose graphic processor units can lead to proper vessel segmentation through automatic processing of 3D volumetric CT data within a reasonable time [7], [8]. This is a crucial step for the quantification of vascular measures in order to aid the diagnosis of vascular diseases [9], [10].

Tortuosity is a measure of twistedness of blood vessels and can increase due to hypertension or vasculopathies [11]–[13]. Tortuosity is also applied in clinical settings to differentiate between benign and malignant tumours [14], [15] or to characterize retinal vascular changes [16]. The most common metric of vascular tortuosity is the “distance metric”, which provides a ratio of the actual vessel path length to the linear distance between its endpoints [17], [18]. This metric has been used to characterize tortuosity of tumors using 3D animal microCT [19], [20]. Another parameter to measure the complexity of the lung vascular tree is the fractal dimension. A fractal is a self-similar object over different scales [21]. The complexity of a fractal object can be measured by the fractal dimension (FD) which is a measure of space filling [22]–[24]. This parameter has been used for characterization of the human retina [16], [25] or different tumour entities [26], [27]. Two studies evaluated the fractal dimension of 2D projections of the lung vascular system in patients with PH [28], [29]. Moledina et al. showed that the 2D FD of children suffering from PH negatively correlated with the pulmonary vascular resistance, WHO functional class and 6-min walk distance. Moreover, in their study, decreased FD was associated with poor survival. In the other study [29] an increased 2D FD of the projected pulmonary arteries in PH patients was associated with an increased mPAP.

The purpose of this study was to automatically detect the lung vessels from contrast-enhanced chest CT scans in 3D and quantify their tortuosity and 3D fractal dimension. These measures were compared to patient clinical parameters derived from RHC.

## Patients and Methods

### Ethics statement

The study was approved by the Institutional Review Board of the Medical University of Graz under the number *23*–*356 ex 10/11* and written informed consent was obtained from all patients.

### Patient population

**Figure 1:**
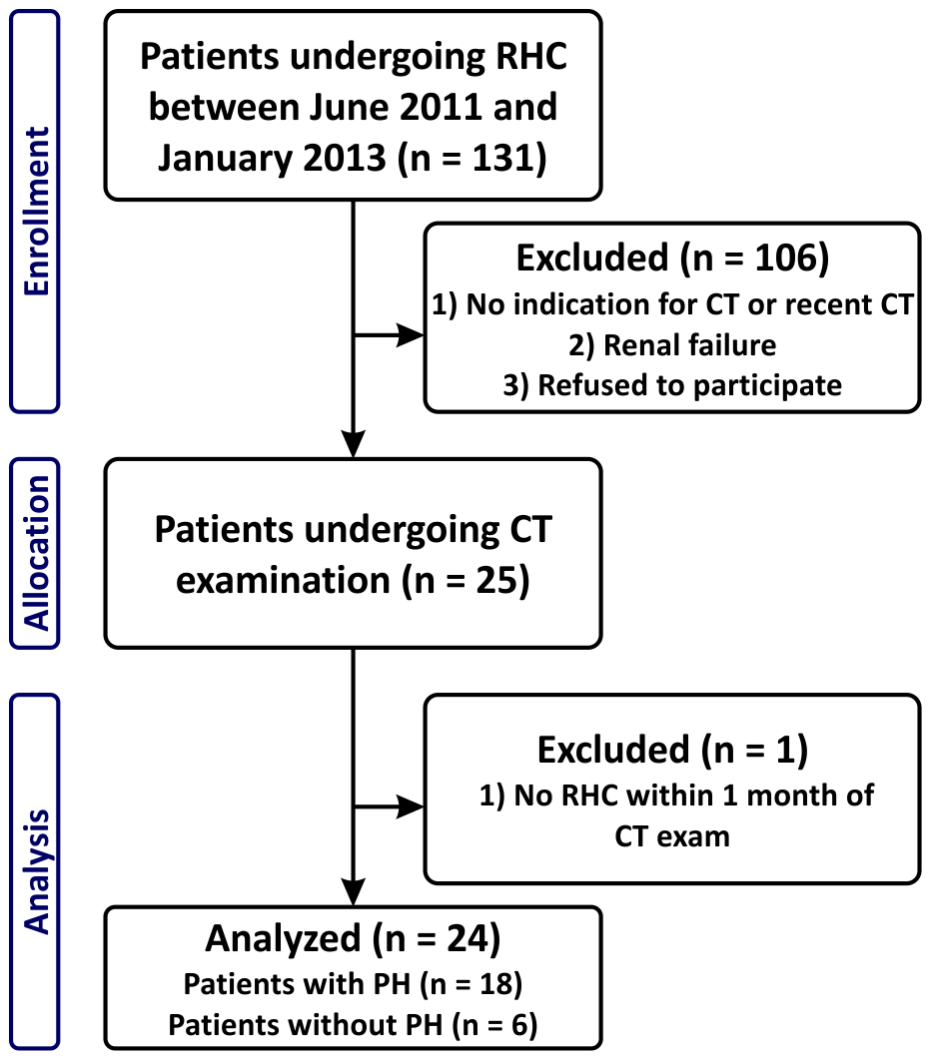
Flowchart of patient recruitment. RHC =  right-sided heart catheterization, CT =  computed tomography, PH =  pulmonary hypertension

All patients undergoing diagnostic or follow-up RHC at the Division of Pulmonology between June 2011 and January 2013 with indication for diagnostic contrast-enhanced thoracic CT were included (Figure 1). Both, patients with and without PH were included. Exclusion criteria were renal failure (creatinin > 1.3 mg/dl), known adverse reactions against iodinated CM, a recent diagnostic CT, more than 1 month between RHC and CT examination, pregnancy and missing written informed consent.

### Examinations

RHC was performed on all patients for diagnostic or follow-up reasons by the same medical personal with 8 years of experience. A 7-F quadruple-lumen, balloon-tipped, flow-directed Swan-Ganz catheter (Baxter Healthcare, Irvine, California) was used in a transjugular approach without transparency.

The thoracic CT examination was performed with a 128-slice dual-energy CT scanner (Somatom Definition Flash, Siemens, Forchheim, Germany). X-ray tube A was set to an acceleration voltage of 100 kV with a reference current time product of 91 mAs_ref_ and tube B to 140 kV with 77 mAs_ref_ together with a 0.4 mm tin filter. Pitch was set to 1.0 and an automatic exposure control was used to reduce the X-ray dose. 40 ml of non-ionic contrast material (Ultravist 370 mg/ml iodine, Bayer Schering Pharma Diagnostic Imaging, Leverkusen, Germany) were injected into an arm vein at 5 ml/s with an automatic power injector (CT-Injector Ohio Tandem, Ulrich, Ulm, Germany). This was followed by a 21 ml saline chaser injected at the same rate. For timing of the scan a test bolus was used. The CT examination protocol was set before the first examination. The results of the RHC were known at the CT examination in order to set time intervals for the test bolus examination [30]. The images were reconstructed with 0.6 mm slice thickness using a medium-soft kernel (D30f), anonymised and transferred to an independent workstation. Dual-energy CT was used to determine the blood flow in the lung parenchyma but is not evaluated in this study. Therefore, for the analysis the mixed images from both detectors with a mixture of tube A/tube B of 60%/40% were used, thus resembling typical appearance of a single 120 kV scan.

### Data processing and analysis

#### Vessel segmentation algorithm

**Figure 2:**
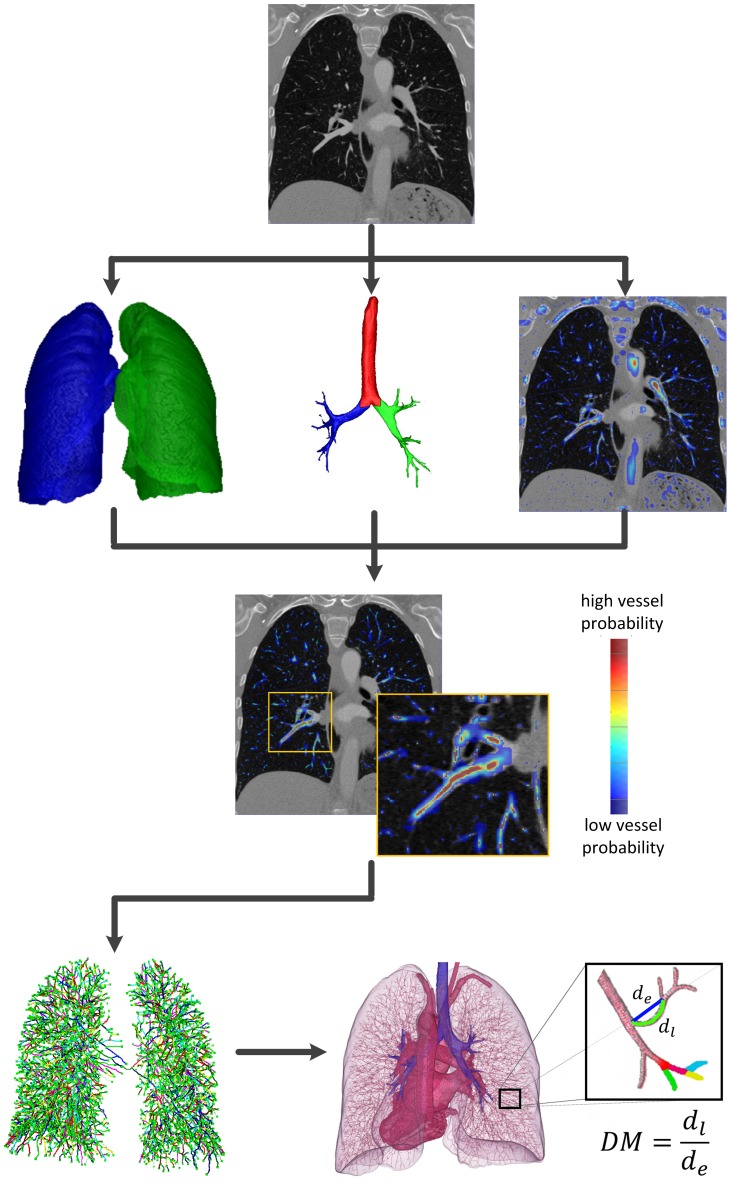
Flowchart of the automatic vessel extraction algorithm. (top) Sample CT image, (2^nd^ row) lung, airway segmentation and the vessel enhancement filter response superimposed on the CT image, (3^rd^ row) vessel enhancement filter response restricted to the region of interest, (bottom row, left) connected centerlines, (bottom row, right) 3D rendering of the lung vessel centerlines. Inset shows the computation of distance metric (DM). The sum of distances along the 3D points of the vessel is divided by the length of the straight path between the two endpoints (first and last 3D point of the vessel segment).

The lung vessels were segmented fully automatically with a validated algorithm developed in-house [31]. The flowchart of the algorithm is shown on Figure 2. The inputs for the algorithm were contrast enhanced thoracic CT scans. In a preprocessing step the CT image was smoothed using an edge preserving total variation based denoising filter [32]. The lung was then segmented by grey-level thresholding [33] followed by morphological closing operations. The bronchi were segmented automatically by detecting a point inside the trachea, applying an iterative 3D-region growing algorithm and splitting the result at the carina. Bronchi segmentation was used to separate the lung segmentation into left and right lung and to remove the bronchial walls from the target processing region. This was necessary since the intensity contrast between the airway border and the vessels is low, therefore, incorrect detection of the blood vessels could occur.

The vessel-enhancement filter (VEF) was using the eigenvectors and eigenvalues of a Hessian matrix, which give information about the local image structure, to detect tubular structures. To improve accuracy and robustness, at each position an offset-medialness boundary measure perpendicular to the estimated vessel direction was evaluated [34] and combined with the gradient magnitude at the current position [35]. This led to the final VEF response (Figure 2). After non-maxima suppression of the VEF response, centerlines of the vessels were detected and connected by applying a shortest path algorithm [36]. The 3D rendering of the resulting centerlines is presented in the bottom part of Figure 2. Therefore, we obtained vessel segments for arteries and veins combined which were counted and used for analysis.

#### Calculation of vessel tortuosity

A measure of vascular tortuosity is the “distance metric”, which provides a ratio of the actual vessel path length to the linear distance between its endpoints. We identified all vessel segments inside the lung, where a vessel segment is defined as the path between either two branching points or between a branching point and an end point. For each segment we computed the 3D length of it and divided it by the Euclidean distance between its endpoints (Figure 2, bottom row inset). The result is a dimensionless number reflecting the bending of the vessel segment. The mean value of the distance metric from all vessel segments was used for analysis.

#### Calculation of 3D FD with the box counting method

The fractal dimension of the connected vessel centerlines was computed by applying a 3D extension of the well-validated box counting method [37]. Box counting consists of dividing the image volume with the vessel centerlines into a grid of equal cubes with size δ, and counting the number of cubes containing part of the vessel centerlines. This process was repeated for different cube sizes (from one pixel up to 100 pixel side length). The number of cubes containing vessels is plotted against the cube size (δ) in a double logarithmic plot (Figure S1). The fractal dimension is equivalent to the slope of the fitted line. To account for limitations in resolution, only the linear part (Figure S1, red dots) was used for line fitting. This was carried out by iteratively discarding the data points from the small δ range from the linear fit until a good fit was achieved. Subsequently, the data points from the large δ range were discarded while still keeping the good correlation. On average 30 points were used for the fit.

### Statistical analysis

Statistical analysis was performed in GraphPad Prism (Version 5.04, La Jolla, California). Correlations between CT and RHC derived parameters were calculated with linear regression and Spearman correlation. Differences between PH and non-PH patients were determined with t-test, whereas differences between patients' WHO functional classes were assessed by non-parametric analysis of variances (ANOVA, Kruskal-Wallis test). Receiver-operating analysis was used to assess the conclusiveness of the parameters to determine the presence of PH and to calculate optimal cut-off values. P-values (p) ≤0.05 were considered as significant.

## Results

Twenty-four consecutive patients (female:male = 14∶10) were included in this study (Figure 1). Patient characteristics are summarized in Table 1. The patient group consisted of 18 patients with PH (*n* = 4 with idiopathic pulmonary arterial hypertension (IPAH), *n* = 5 with associated pulmonary arterial hypertension (APAH), *n* = 2 with PH associated with lung disease, *n* = 7 with chronic thromboembolic pulmonary hypertension) and 6 patients without PH (*n* = 4 with systemic sclerosis, *n* = 1 with interstitial lung disease and *n* = 1 patient, who presented without PH after pulmonary endarterectomy). The CT examinations were indicated to exclude relevant lung parenchymal diseases (*n* = 17 patients), to control lung fibrosis (*n* = 4) and because of a suspected progression of scleroderma lung disease (*n* = 3). The CT examination was carried out within a median of 1 day (range 1–18 days) from a diagnostic or follow-up RHC. No change in therapy occurred during this time. There were no complications during RHC or during CT examination. The average effective dose of the thoracic CT scan according to ICRP 103 guidelines was 3.6±1.4 mSv (dose length product: 180±70 mGycm) [38], [39].

**Table 1:** Patient characteristics.

Patient characteristics	All patients	No PH	PH
**Number of patients**	24	6	18
**Female/male**	14/10	4/2	10/8
**WHO class (I/II/III/IV)**	2/14/8/0	1/5/0/0	1/9/8/0
**Age [years]**	60±13 (27–76)	59±8 (50–71)	61±15 (27–76)
**BSA [m^2^]**	2.0±0.3 (1.6–2.9)	2.0±0.2 (1.8–2.3)	2.0±0.3 (1.6–2.9)
**mPAP [mmHg]**	36±15 (14–66)	17±2 (14–20)	43±12 (26–66)
**PAWP [mmHg]**	9±3 (3–15)	8±2 (5–11)	9±3 (3–15)
**CO [l/min]**	4.5±1.2 (2.9–7.8)	5.5±1.5 (4.3–7.8)	4.2±0.9 (2.9–5.7) *
**PVR [dynscm^−5^]**	540±370 (80–1420)	110±20 (80–130)	680±320 (230–1420) ***
**AVDO_2_ [vol%]**	4.9±1.0 (2.4–6.4)	4.0±0.5 (3.3–4.7)	5.2±1.0 (2.4–6.4) **
**art SO_2_ [%]**	94±2 (89–98)	96±1 (95–98)	93±2 (89–98) **
**ven SO_2_ [%]**	68±7 (50–84)	74±2 (71–76)	66±7 (50–84)*

Data are presented as mean ± SD (range). The significance was tested with t-test.

PH: pulmonary hypertension, BSA: body surface area after Dubois and Dubois, mPAP: mean pulmonary arterial pressure, PAWP: pulmonary artery wedge pressure, CO: cardiac output, PVR: pulmonary vascular resistance, AVDO_2_: arterial-venous difference in oxygen content, art SO_2_: arterial oxygen saturation, ven SO_2_: venous oxygen saturation, */**/***: significant difference between PH and non-PH patients (p<0.05/0.01/0.001).

The automatic vessel segmentation algorithm was successful in identifying right and left lung lobes, trachea and bronchi in all cases. The number of vessel segments was on average 12427±3508 (range 5922–20434) and was not correlated with the disease, age, body surface area (BSA) or the hemodynamic parameters (Table 2 and 3; Figure S2).

**Table 2:** Values of distance metric, fractal dimension and number of vessel segments.

	All patients (n = 24)	No PH (n = 6)	PH (n = 18)
**Distance metric**	1.224±0.019 (1.199–1.273)	1.208±0.009 (1.199–1.223)	1.230±0.019 * (1.202–1.273)
**Fractal dimension**	2.35±0.06 (2.21–2.44)	2.37±0.08 (2.21–2.43)	2.34±0.05 (2.27–2.44)
**Nr. vessel segments**	12427±3508 (5922–20434)	11719±3041 (5922–14642)	12392±3616 (7815–20434)

Data are presented as mean ± SD (range). The significance was tested with t-test.

PH: pulmonary hypertension, *: significant difference between PH and non-PH patients (p<0.05).

**Table 3:** Correlations with clinical parameters (Spearman r and p-value) for n = 24 patients.

r (p)	Distance metric	Fractal dimension	No. vessel segments
**mPAP**	**0.60** (0.002)	**−0.30** (0.15)	**0.01** (0.97)
**PVR**	**0.59** (0.002)	**−0.34** (0.10)	**−0.11** (0.61)
**AVDO_2_**	**0.54** (0.007)	**−0.37** (0.07)	**0.08** (0.68)
**art SO_2_**	**−0.54** (0.006)	**0.47** (0.02)	**0.15** (0.48)
**ven SO_2_**	**−0.68** (0.0002)	**0.38** (0.07)	**−0.06** (0.77)
**Age**	**−0.12** (0.57)	**−0.24** (0.24)	**−0.37** (0.07)
**BSA**	**−0.04** (0.83)	**0.03** (0.89)	**0.08** (0.68)

mPAP: mean pulmonary arterial pressure, PVR: pulmonary vascular resistance, AVDO_2_: arterial-venous difference in oxygen content, art SO_2_: arterial oxygen saturation, ven SO_2_: venous oxygen saturation, BSA: body surface area after Dubois and Dubois.

**Figure 3:**
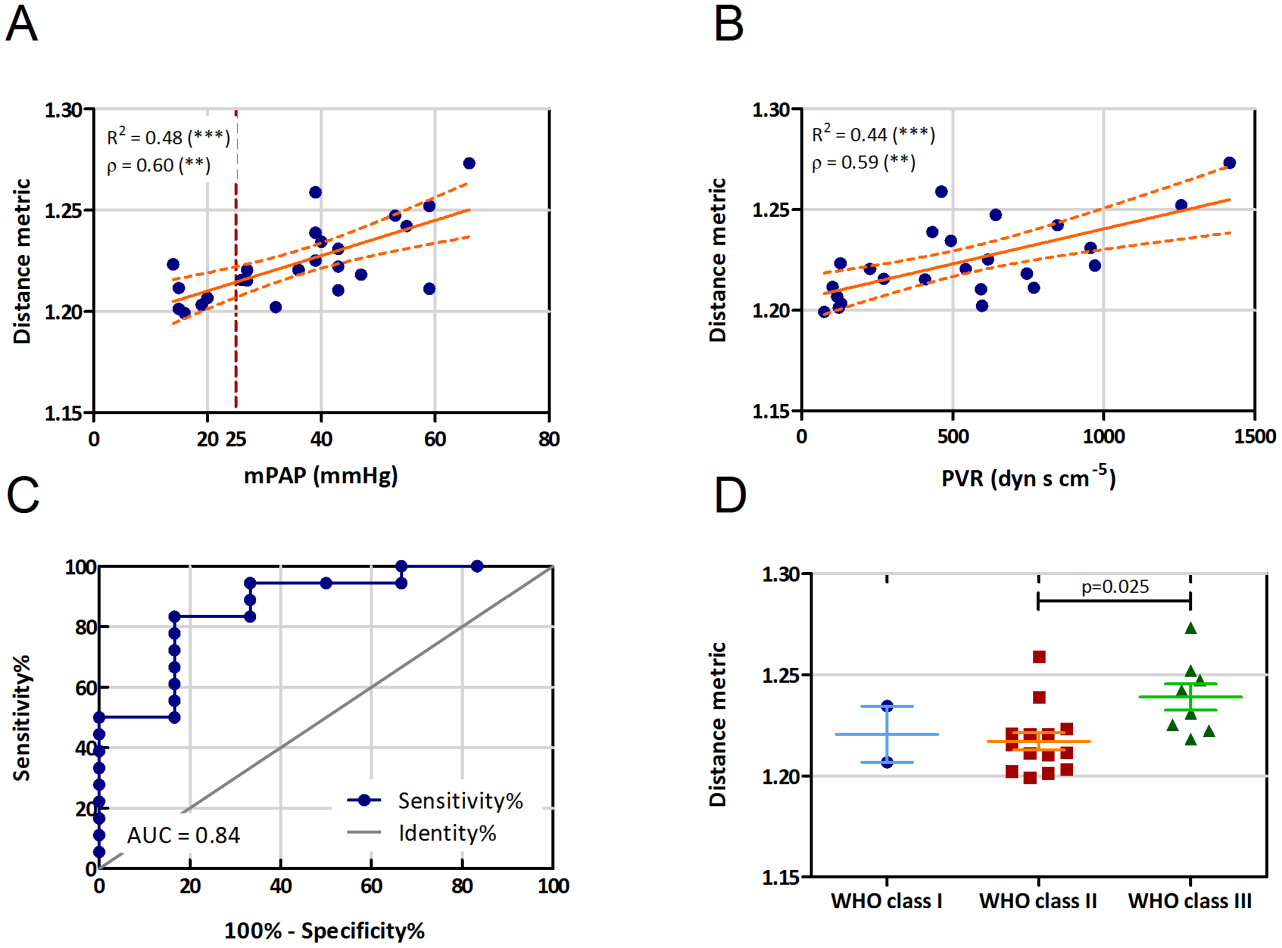
Correlation of distance metric with patient clinical parameters. Correlation of distance metric with (A) mean pulmonary arterial pressure (mPAP), and (B) pulmonary vascular resistance (PVR; R =  linear correlation coefficient, r =  Spearman correlation coefficient, ** p<0.01, *** p<0.001). (C) Receiver-operating curve for DM determining mPAP >25 mmHg (AUC: area under the curve). (D) Distribution of distance metric according to the WHO classification of the patients. (solid lines represent mean and standard error of mean; p value shows significant difference between WHO class II and III).

There was a significant difference between the distance metric of patients with and without PH (1.230±0.019 vs 1.208±0.009, Table 2). Moreover, we found a correlation between mean pulmonary arterial pressure (mPAP) and the distance metric of ρ = 0.60 (Figure 3a). Further, there was a significant correlation with the pulmonary vascular resistance (PVR; ρ = 0.59, Figure 3b). The receiver operating curves showed a discriminative power of this parameter. The area under the curve (AUC) was 0.84 (sens/spec 83%/83%, for a distance metric > 1.213; Figure 3c). Moreover, there was a significant association of distance metric with WHO functional class (p = 0.025 between WHO class II and III, Figure 3d).

**Figure 4:**
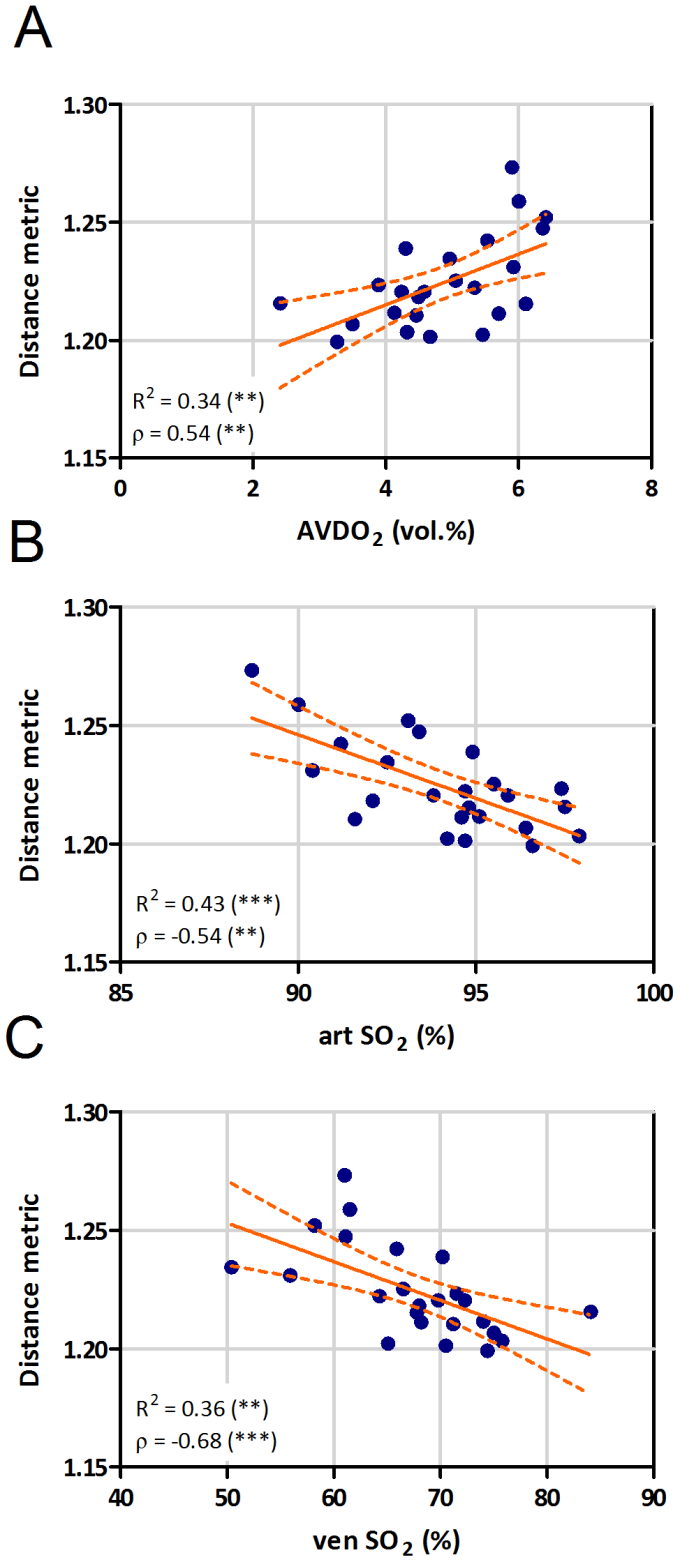
Correlation of distance metric with oxygen exchange parameters. Correlation of distance metric with arterio-venous difference in oxygen content (AVDO_2_, A), arterial (art SO_2_, B) and venous (ven SO_2_, C) oxygen saturation (R =  linear correlation coefficient, ρ = Spearman correlation coefficient, ** p<0.01, *** p<0.001).

Besides the main diagnostic parameters, distance metric significantly correlated with other hemodynamic parameters, like the difference between arterial and venous oxygen content (AVDO_2_, ρ = 0.54, Figure 4a) or arterial (artSO_2_, ρ = −0.54, Figure 4b) or venous oxygen saturation (venSO_2_, ρ = −0.68, Figure 4c). In order to show the specificity of this tortuosity parameter, we correlated the distance metric with age and BSA. None of these parameters showed a significant correlation (Table 3).

**Figure 5:**
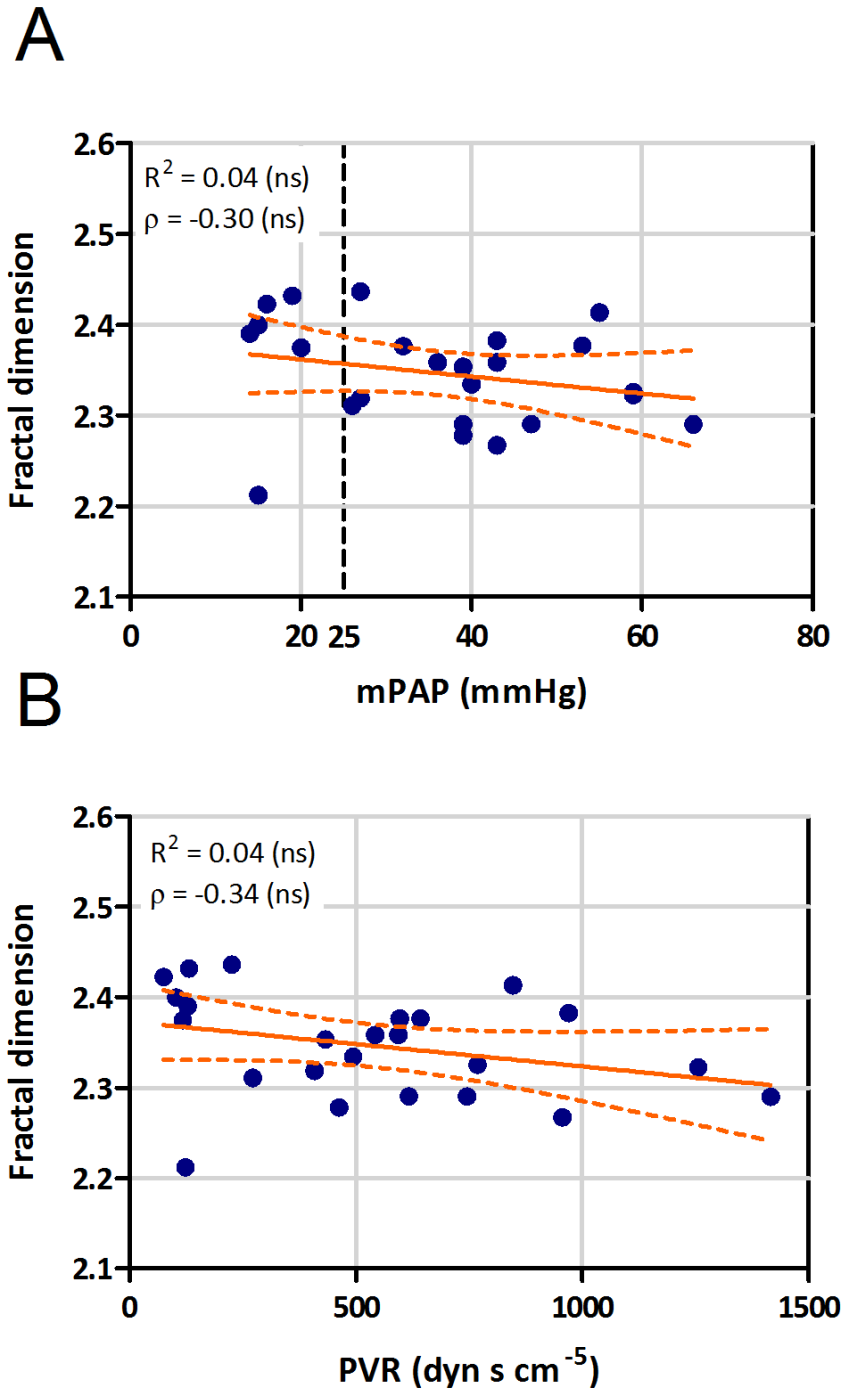
Correlation of fractal dimension with clinical parameters. Correlation of 3D fractal dimension (FD) with (A) mean pulmonary arterial pressure (mPAP), and (B) pulmonary vascular resistance (PVR; R =  linear correlation coefficient, ρ =  Spearman correlation coefficient, ns - not significant).

**Figure 6:**
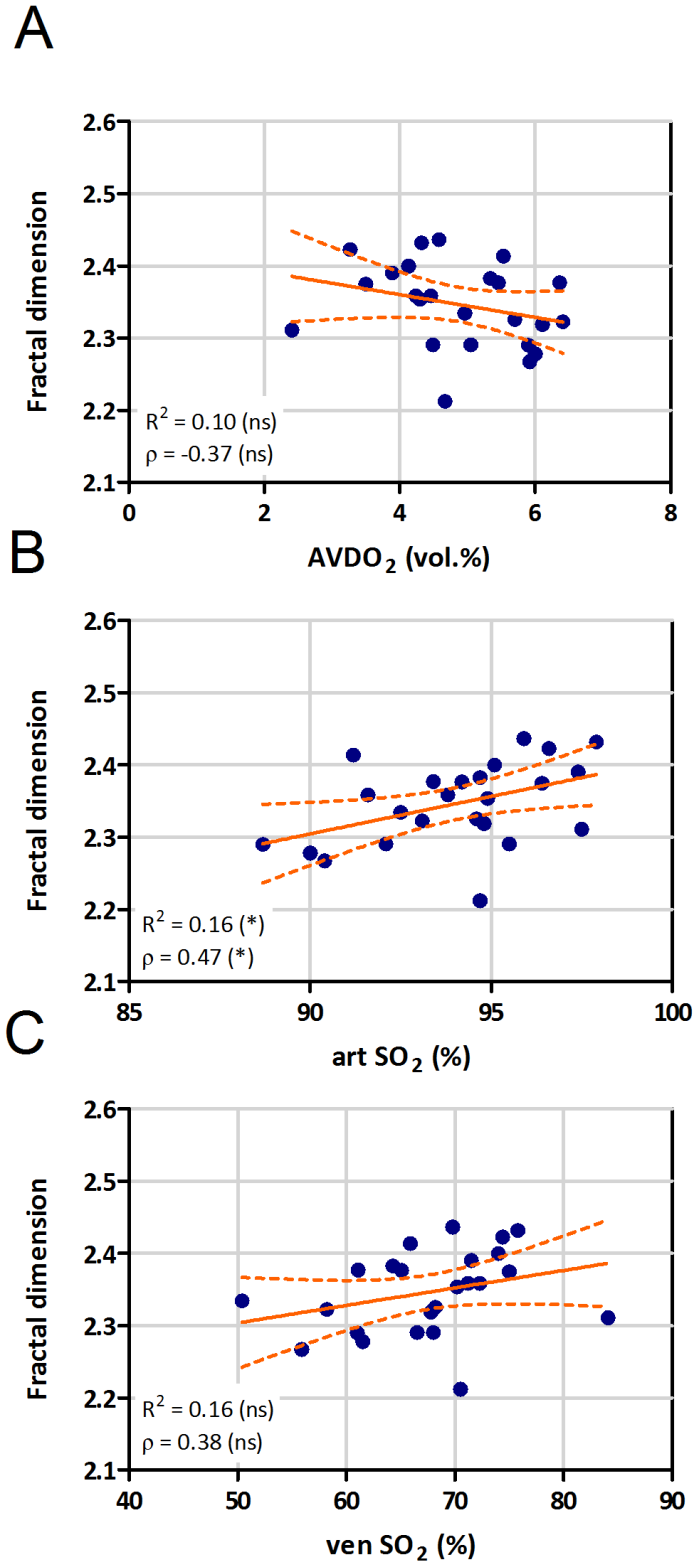
Correlation of fractal dimension with oxygen exchange parameters. Correlation of 3D fractal dimension with arterio-venous difference in oxygen content (AVDO_2_, A), arterial (art SO_2_, B) and venous (ven SO_2_, C) oxygen saturation (R =  linear correlation coefficient, ρ =  Spearman correlation coefficient, * p<0.05, ns - not significant).

The mean value of the 3D fractal dimension in our patient cohort was 2.35 (range 2.21–2.44, Table 2), which is in good agreement with previously reported values from similar studies [27]. In contrast, there was no significant correlation of 3D FD either with mPAP or with PVR (Figure 5a, b). 3D fractal dimension was negatively correlated with AVDO_2_ (ρ = −0.37), and positively with artSO_2_ (ρ = 0.47) and venSO_2_ (ρ = 0.38), but these correlations were weak and significant only for artSO_2_ (Figure 6). 3D fractal dimension was not associated with WHO functional class (Figure S3).

Neither the distance metric, nor the 3D fractal dimension showed significant differences between different forms of pulmonary hypertension (Figure S4).

## Discussion

In this pilot study we showed that vessel tortuosity, determined by our automatic vessel extraction algorithm using routine contrast enhanced thoracic CT scans, is correlated with mean pulmonary arterial pressure, pulmonary vascular resistance as well as measures of pulmonary gas exchange. There was a significant increase of the mean distance metric (tortuosity readout) of patients with PH as compared to patients at risk for PH with mPAP<25mmHg, suggesting that this measure could be suitable for PH screening. In contrast, we did not observe correlations of 3D fractal dimension with pulmonary hemodynamics or gas exchange of these patients.

The automatic vessel detection algorithm allows physical characterization of the pulmonary vessel structure. The total number of vessel segments detected by our algorithm is in the range of ten thousands. The number of vessel segments extracted from the CT dataset did not correlate with any relevant clinical parameter (mPAP, PVR, AVDO_2_, artSO_2_ or venSO_2_), suggesting that pruning of pulmonary vessels either affects only smaller vessels or this phenomenon was not relevant for our patient cohort. Moreover, the absence of correlation shows the robustness of the vessel detection algorithm, and that the other readouts are not influenced by the number of detected vessels.

We applied an advanced 3D method to analyse fractal dimension of the lung vascular tree. This technique gives a quantification of the space filling, providing direct readout for 3 dimensional branching of the tree. We did not find any significant correlation of 3D fractal dimension with the hemodynamic parameters and just a moderate positive correlation with arterial oxygen saturation. This is in contrast to a recent investigation, where fractal dimensions were correlated with PVR index, WHO functional class and 6-min walk distance [28]. However, in that study, very young patients (mainly children) were investigated. Pulmonary hypertension could have a different effect on the vascular anatomy when it develops at a very young age, as if it would develop during adulthood, like in our patients. Additionally, for the pulmonary vessel extraction, that study applied a threshold-based region-growing algorithm followed by skeletonization of the vessels, whereas we used a vessel enhancement filter based automatic extraction algorithm, which allows the detection of smaller vessels with lower contrast without the risk of wrongly segmenting parts of the lung parenchyma with higher density. Another study reported increased FD of PH patients [29]. However, their results might be influenced by the 2 dimensional maximum intensity projections used. As we did not observe any correlations of 3D fractal dimension with the main hemodynamic parameters in our patient cohort, we concluded that this measure may not be suitable for detection or explanation of PH in adult patients.

In the systemic circulation it is a well-established fact that systemic arterial hypertension is associated with tortuous systemic arteries [40], [41]. Vascular morphology has been used as diagnostic parameter and for quantification of disease severity in several studies. It was shown that calculating the tortuosity of the brain vessels allows for a distinction between malignant and benign brain tumors [42]. Similarly, the tortuosity of retina vessels was a good measure for vascular malformations in Fabry disease [16]. Although, the presence of tortuous vessels in the lung is considered as a frequent finding in PH [5], [43]–[45], a comprehensive quantification of pulmonary vessel tortuosity, particularly using an automated method, was not presented so far. In pediatric PH patients a significant correlation of the vessel tortuosity (measured as a 3 scale radiological score) with mPAP and PVR was reported [5]. We observed a significant correlation of distance metric with mean pulmonary arterial pressure, pulmonary vascular resistance and measures of gas exchange. Furthermore, there was a significant association of distance metric with WHO functional class, suggesting an increase in tortuosity with increase in disease severity. Moreover, in our adult patient cohort, distance metric, as a measure of tortuosity, showed a good discriminative power between patients with and without PH. The area under the curve of the ROC analysis of 0.84 is similar to the value of 0.86 reported by Janda et al. in their meta-analysis on the diagnostic accuracy of echocardiography [46]. However, a meaningful comparison of the diagnostic accuracy of these two non-invasive methods would require a head-to-head comparison in a larger patient cohort.

Altogether, these results suggest that a non-invasive thoracic CT examination can provide estimates of important parameters derived from an invasive right-sided heart catheterisation. Since there is no user intervention necessary, our algorithm can be run on every thoracic CT scan of patients with an unknown lung disease without additional workload for the radiologist or technician. This might help to non-invasively identify patients with manifest pulmonary hypertension. Additionally, this method could be applied to characterize and better understand gas exchange abnormalities in patients with known pulmonary hypertension.

### Limitations

One of the limitations of this pilot study is the small number of patients, allowing only a preliminary conclusion, despite considering a wide range of diseases. An adequately powered prospective study is currently under way to determine the benefits and drawbacks of this method. Another limitation is the number of vessels accessible by CT imaging. The human lung includes hundreds of thousands of vessels. Huang et al. found a total of 15 generations of vessels between the main pulmonary artery and the capillaries, with diameters from 15 mm down to 0.02 mm [47]. In our CT images we could only detect vessels down to a diameter of approximately 2 mm; smaller vessels cannot be detected due to scanner resolution and the partial volume effect. Our algorithm detects vessel segments regardless of whether they are arteries or veins. Since the increased pressure is confined to the arterial vasculature in patients with pulmonary arterial hypertension, we expect that discrimination between arteries and veins would further improve the diagnostic value of our measures. Such an algorithm, capable of distinguishing arteries and veins, was recently published e.g. by Park et al. [48]. As a further limitation, due to radiation exposure, we could not test the repeatability of the method. This would have been necessary to determine the robustness of the results. However, the correlations with many important parameters of pulmonary blood flow and gas exchange suggest a high degree of reliability of the measurements.

## Conclusion

Vessel tortuosity derived from thoracic CT by automatic 3D extraction of the pulmonary vessels is correlated with pulmonary arterial hemodynamics and gas exchange. This non-invasive method may help understanding the impact of pulmonary vascular changes for hemodynamics and gas exchange, and may provide a screening tool for pulmonary hypertension. Prospective validation of our method in a larger patient cohort is warranted.

## Supporting Information

Figure S1 Double logarithmic plot of the number of cubes (N_δ_) against the cube size (δ) for a representative patient. For linear fitting only the linear part of the data (red crosses) was used. The slope of the fitted line (green) corresponds to the fractal dimension (FD).
(TIF)

Figure S2 Correlation of number of vessel segments with (A) mean pulmonary arterial pressure (mPAP), (B) pulmonary vascular resistance (PVR), (C) arterio-venous difference in oxygen content (AVDO_2_), (D) arterial (art SO_2_) and (E) venous (ven SO_2_) oxygen saturation (R =  linear correlation coefficient, r =  Spearman correlation coefficient, ns - not significant). (F) Distribution of the number of vessel segments according to the WHO classification of the patients (solid lines represent mean and standard error of mean).
(TIF)

Figure S3 Distribution of 3D fractal dimension according to the WHO classification of the patients (solid lines represent mean and standard error of mean).
(TIF)

Figure S4 Distribution of (A) distance metric (DM) and (B) fractal dimension based on disease subtype (PH: pulmonary hypertension, IPAH: idiopathic pulmonary arterial hypertension, APAH: pulmonary arterial hypertension associated with risk factors or conditions, PH-LD: pulmonary hypertension associated with lung disease, CTEPH: chronic thromboembolic pulmonary hypertension).
(TIF)

Checklist S1 STARD checklist for reporting of studies of diagnostic accuracy.
(DOC)
