# Supplementary figures and images for: Quantification of Tortuosity and Fractal Dimension of the Lung Vessels in Pulmonary Hypertension Patients
Source: PLoS One. 2014 Jan 31;9(1):e87515. doi: 10.1371/journal.pone.0087515 (PMC3909124; doi:10.1371/journal.pone.0087515)

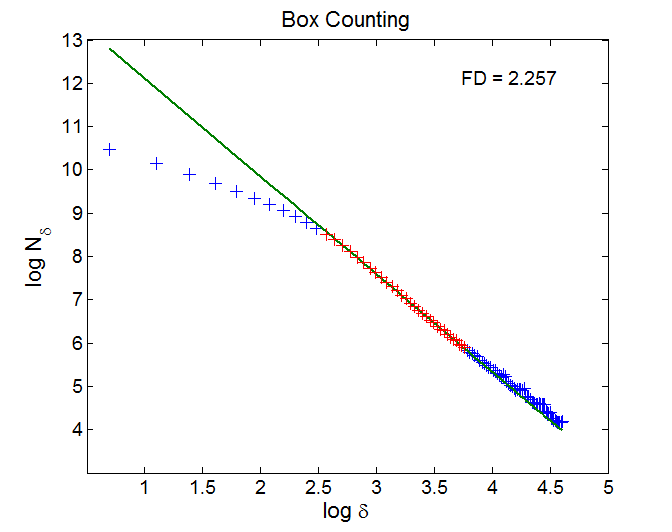

Supplement: Figure S1 — Double logarithmic plot of the number of cubes (Nδ) against the cube size (δ) for a representative patient. For linear fitting only the linear part of the data (red crosses) was used. The slope of the fitted line (green) corresponds to the fractal dimension (FD). (TIF) [file pone.0087515.s001.tif]

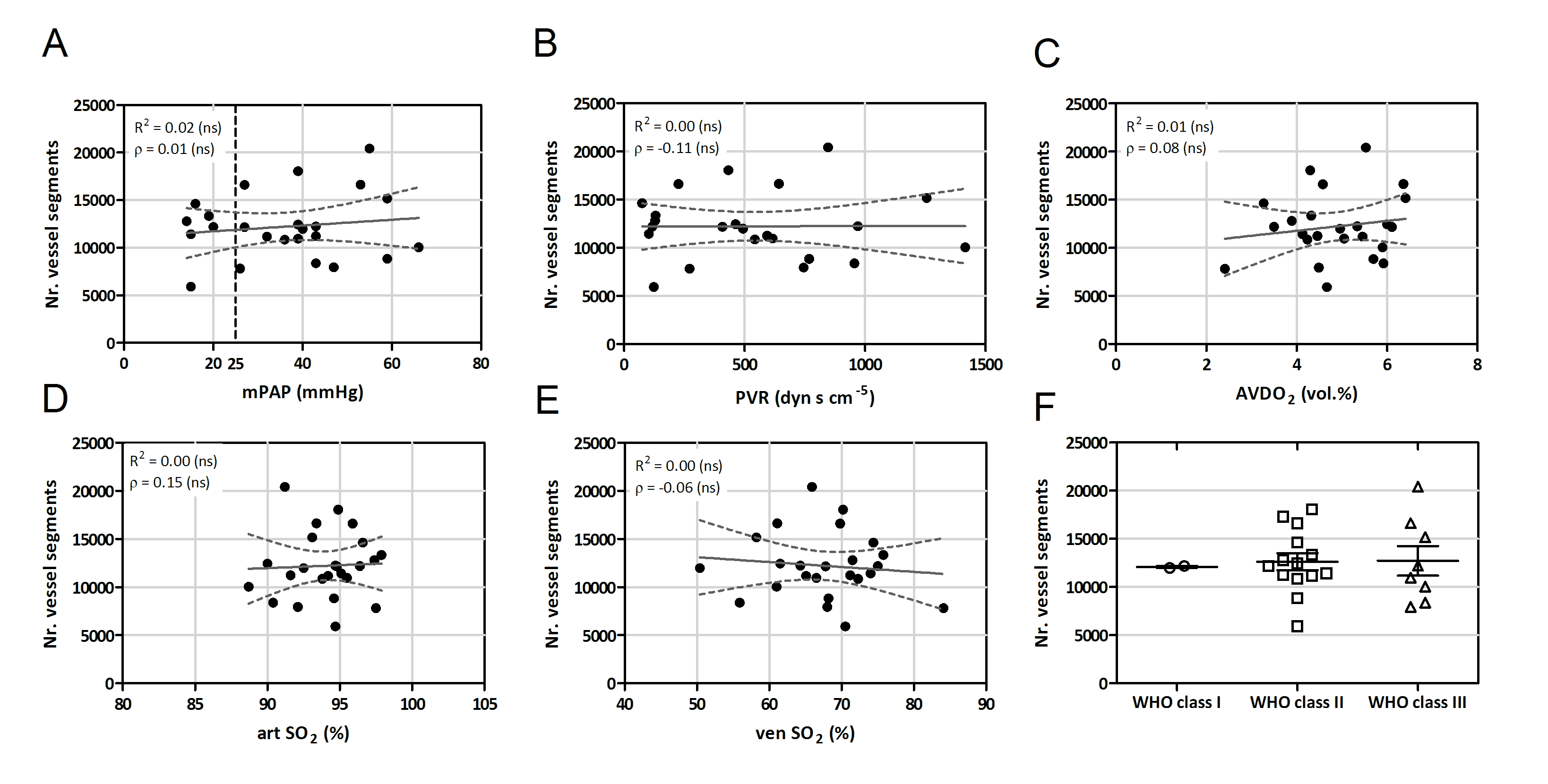

Supplement: Figure S2 — Correlation of number of vessel segments with (A) mean pulmonary arterial pressure (mPAP), (B) pulmonary vascular resistance (PVR), (C) arterio-venous difference in oxygen content (AVDO2), (D) arterial (art SO2) and (E) venous (ven SO2) oxygen saturation (R = linear correlation coefficient, r = Spearman correlation coefficient, ns - not significant). (F) Distribution of the number of vessel segments according to the WHO classification of the patients (solid lines represent mean and standard error of mean). (TIF) [file pone.0087515.s002.tif]

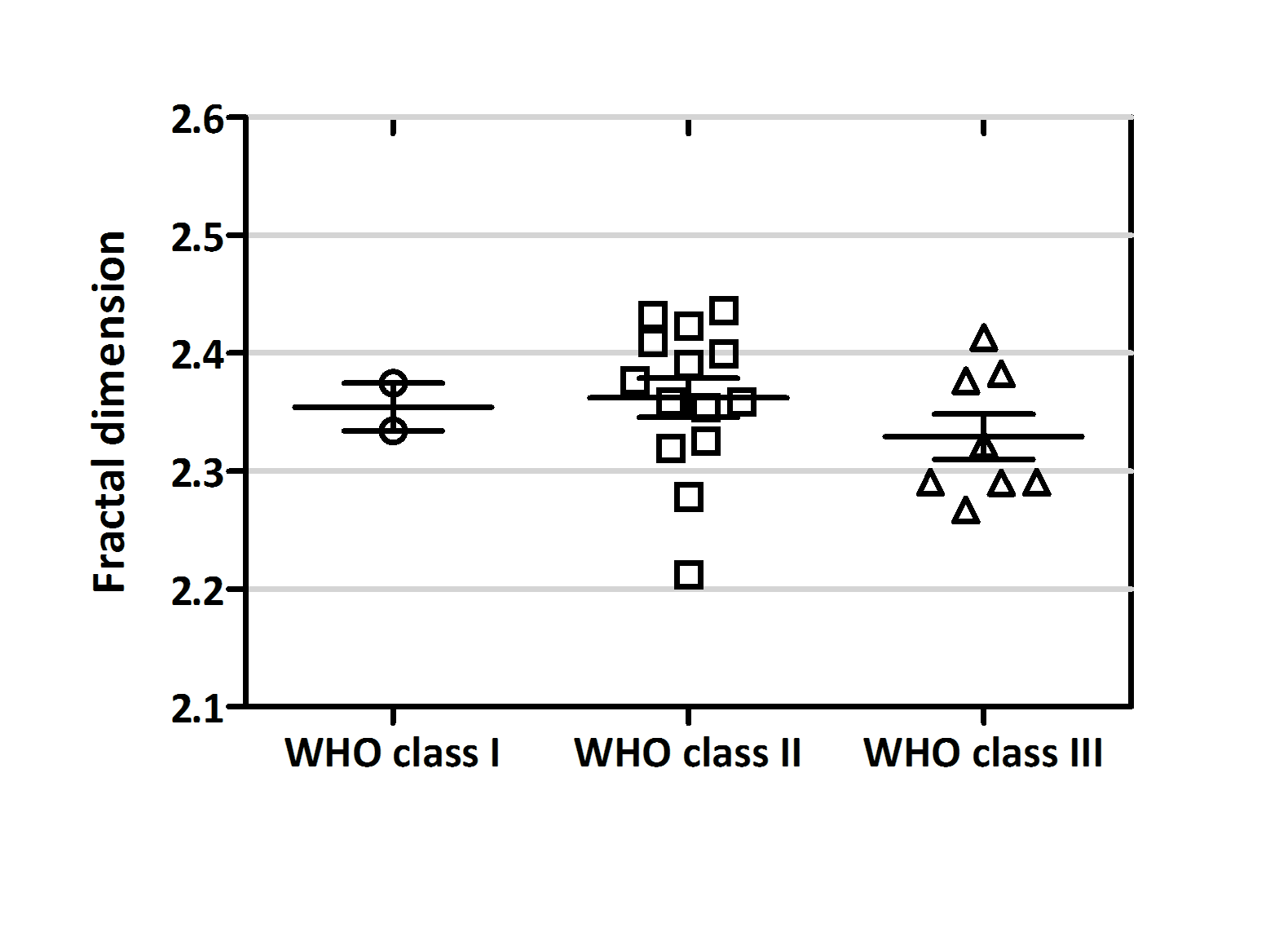

Supplement: Figure S3 — Distribution of 3D fractal dimension according to the WHO classification of the patients (solid lines represent mean and standard error of mean). (TIF) [file pone.0087515.s003.tif]

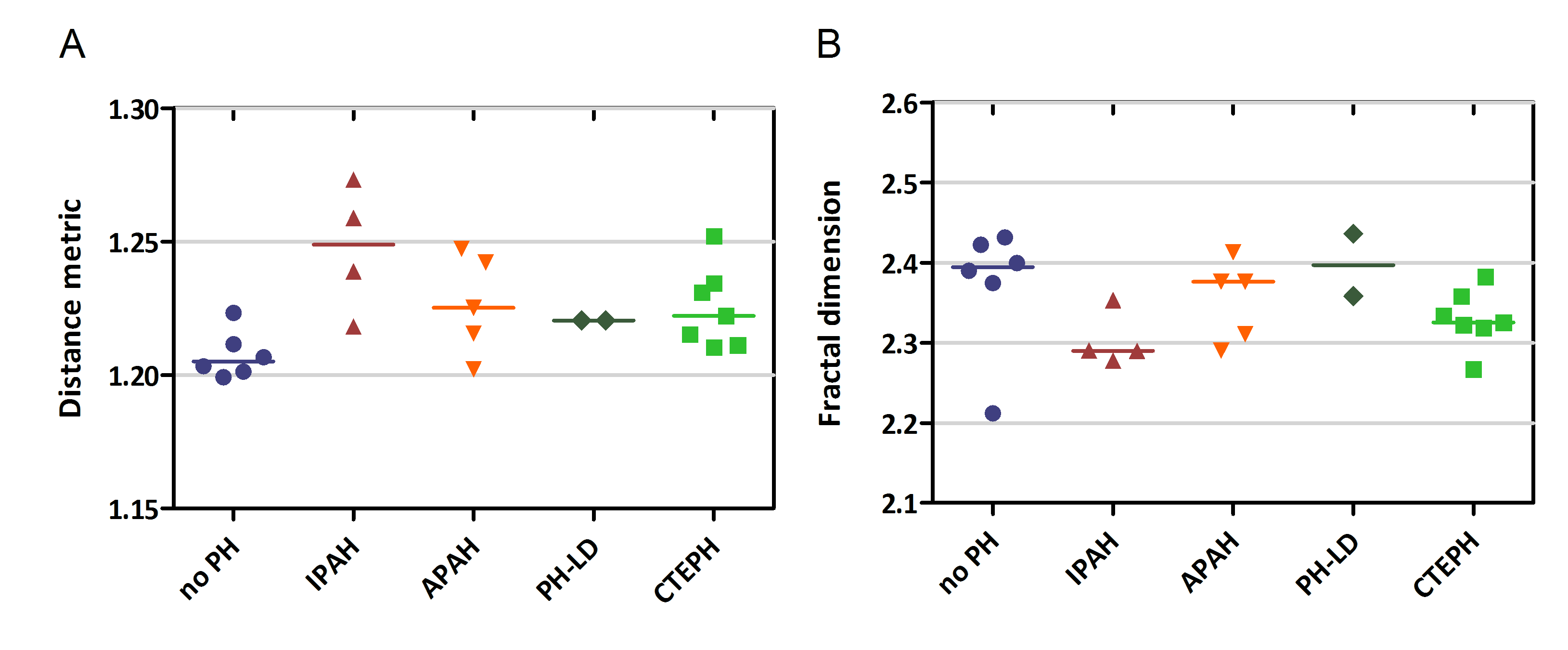

Supplement: Figure S4 — Distribution of (A) distance metric (DM) and (B) fractal dimension based on disease subtype (PH: pulmonary hypertension, IPAH: idiopathic pulmonary arterial hypertension, APAH: pulmonary arterial hypertension associated with risk factors or conditions, PH-LD: pulmonary hypertension associated with lung disease, CTEPH: chronic thromboembolic pulmonary hypertension). (TIF) [file pone.0087515.s004.tif]
